# Beyond Conventional Organic Electrosynthesis: The Role of Fluorinated Solvents

**DOI:** 10.1021/acselectrochem.4c00129

**Published:** 2024-11-27

**Authors:** Xavier Marset, Salvador Montilla-Verdú, Elio Rico, Néstor Guijarro

**Affiliations:** Institute of Electrochemistry, 16718 Universidad de Alicante, Apdo. 99, 3080 Alacant, Spain

**Keywords:** electrosynthesis, fluorinated solvent, solvent effect, sustainability, organic chemistry

## Abstract

Organic electrosynthesis has emerged as a unique platform for chemical manufacturing owing to not only the use of electricity as a green reagent but also, especially, to its distinct reactivity. While conventional solvents are sought to remain inert and solely provide a liquid environment for the electrochemical process to occur, fluorinated alcohol solvents have been shown to redefine this concept. In fact, the singular properties of these solvents allow them to actively interact with the substrates and reaction intermediates driving dramatic changes in the chemo- and regioselectivity as well as on the reaction yields. Given the rapid permeation of these solvents in the burgeoning field of electro-organic synthesis, this mini-review strives to provide a concise but up-to-date critical revision for the growing community of scientists working at the interface of synthetic chemistry and electrochemistry. Here, the main electrosynthetic transformations where they have been exploited besides their key role in activating certain reaction pathways will be highlighted. Finally, a forward-looking perspective on the more practical evolution and implementation of these systems will be discussed.

## Graphical abstract

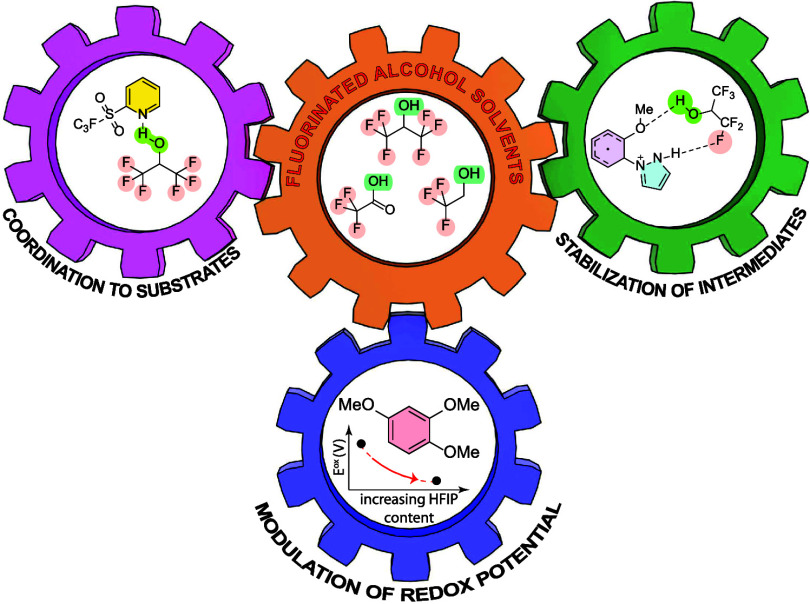

## Introduction

Synthetic organic chemistry is an integral part of several chemical industries, namely pharmaceutical,^1^ agricultural,^2^ petrochemical, and flavors and fragrances,^3^ amongst others. While the combined industrial and academic effort has yielded over 100 million different small molecules, some authors have anticipated over 166 billion small molecules with potential to be lead compounds only in drug development.^4^ In this scenario, enabling new tools capable of simplifying and accelerating the optimization of chemical transformations is crucial to tap into this vast chemical space. Organic electrosynthesis has drawn increasing attention in the last few years as a reliable alternative for conventional organic synthetic protocols wherein redox processes govern the reaction mechanism.^5^ The attractiveness of organic electrosynthesis relies on the prospects of replacing oxidizing or reducing agents by simply electricity. As a result, the cost of the process drops drastically since redox agents are not needed and besides, the work-up is simplified given the reduced possibilities of by-products and of unreacted chemicals.^6^ This, indeed, aligns well with the green chemistry principle of atom efficiency which is set to be the foundation for the next generation of synthetic processes.^7^ Although the field of electro-organic synthesis emerged as a platform to replicate conventional synthetic methodologies, in recent years it has evolved as an independent manufacturing platform with additional degrees of freedom that not only open new gateways to fine-tune the reactivity but, more importantly, bring access to chemical compounds unachievable otherwise.^8^ Broadly speaking, the electrochemical cell consists of two electrodes immersed in an electrolyte that contains the substrates (Figure 1). Variations of this architecture include a third electrode (reference) to control or sense the electrodes’ potential or a membrane to separate both compartments (catholyte and anolyte) to avoid crossover of sensitive species. The reaction is triggered by passing a constant current through the cell or, less often, by applying a certain potential difference between both electrodes. Note that the targeted product could result from the specific oxidation (or reduction) process or the homogeneous reaction between the species generated at the anode and cathode. On the one hand, the prospect of introducing electricity as a reagent brings out unique possibilities regarding the control over the reactivity and the selectivity. For instance, tuning the applied current allows modulation of the rate of the reaction, whereas adjusting the applied potential affords virtually mimicking of the oxidizing or reducing power of any redox reagent, thus providing unprecedented control over the chemoselectivity.^9,10^ On the other hand, expanding further on the components of the device, unlike the electrodes’ composition, which has been largely explored and exploited to improve the reactivity,^11^ the prospects of engineering the solvent for this purpose remains largely overlooked.

**Figure 1:**
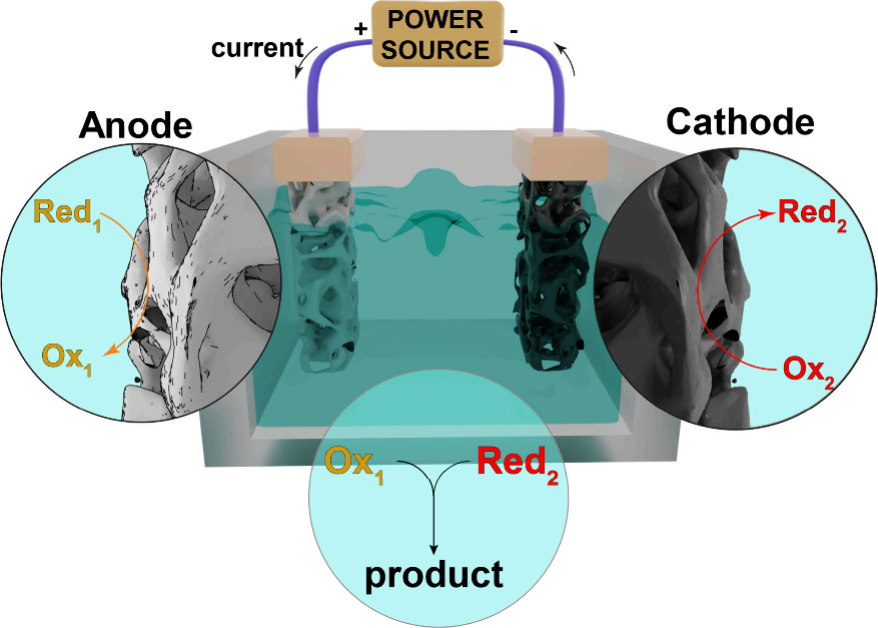
Schematic representation of a single-compartment 2-electrode electrochemical cell. The oxidation (of Red_1_ species) and reduction (of Ox_2_ species) occurring at the anode and cathode gives rise to Ox_1_ and Red_2_ species, respectively. In this scenario, one of them could be the targeted product, while the other reaction will take place as a sacrificial process to sustain the current, or both could be desired products. Likewise, one or both of these species could be precursor(s) that yield the product via homogeneous reaction in the reaction medium.

Generally, the selection of the solvent only fulfills the requirements of solubilizing the chemicals involved in the process and of being electrochemically inert, thus behaving as a mere spectator in the whole process. However, custom-designing solvents to purposely interact with the substrate or intermediates will broaden the versatility of electrosynthesis and provide new strategies to adjust the reactivity.

In recent years, fluorinated alcohol solvents have come into the spotlight as a key ingredient to modulate the electroactivity of a wide range of systems and even activate reactions that otherwise could not occur. This “magical” effect, as coined elsewhere, has been attributed to their distinctive interactions within the reaction media (Figure 2).^12^ Several studies have demonstrated that they could stabilize not only ion pairs, but more surprisingly, cationic intermediates owing to their high polarity as well as radicals.^13^ This distinct high polarity combined with their low nucleophilicity has also been invoked to explain the activation of certain reagents, like halides.^14^ On the other hand, the possibility to coordinate via hydrogen bonding to certain substrates or intermediates has been brought up to account for the unique regioselectivity that can be achieved in the presence of these solvents.^15^ Likewise, the distinct interactions with the solvent can dramatically change the redox potentials of the substrates,^16^ and hence, the reactivity. In view of the unique properties and benefits that these solvents display, it is not unreasonable to think that their permeation into the field of electrosynthesis will grow, setting the foundations for a new generation of active solvents.^17^ This mini-review aims at providing a critical revision on the last 5-year progress on the use of fluorinated solvents for organic electrosynthesis given the fast pace of development. This contribution will cover (i) a brief discussion on the properties of most common fluorinated alcohol solvents; followed by (ii) a comprehensive classification of the key synthetic reactions wherein these solvents display an active role, including the interplay and specific benefits in the reaction media; (iii) as well as a brief discussion on the practicality of fluorinated solvents; and finally, (iv) a conclusion and outlook wherein a forward-looking perspective on the implementation and impact of these emerging solvents will be discussed.

**Figure 2:**
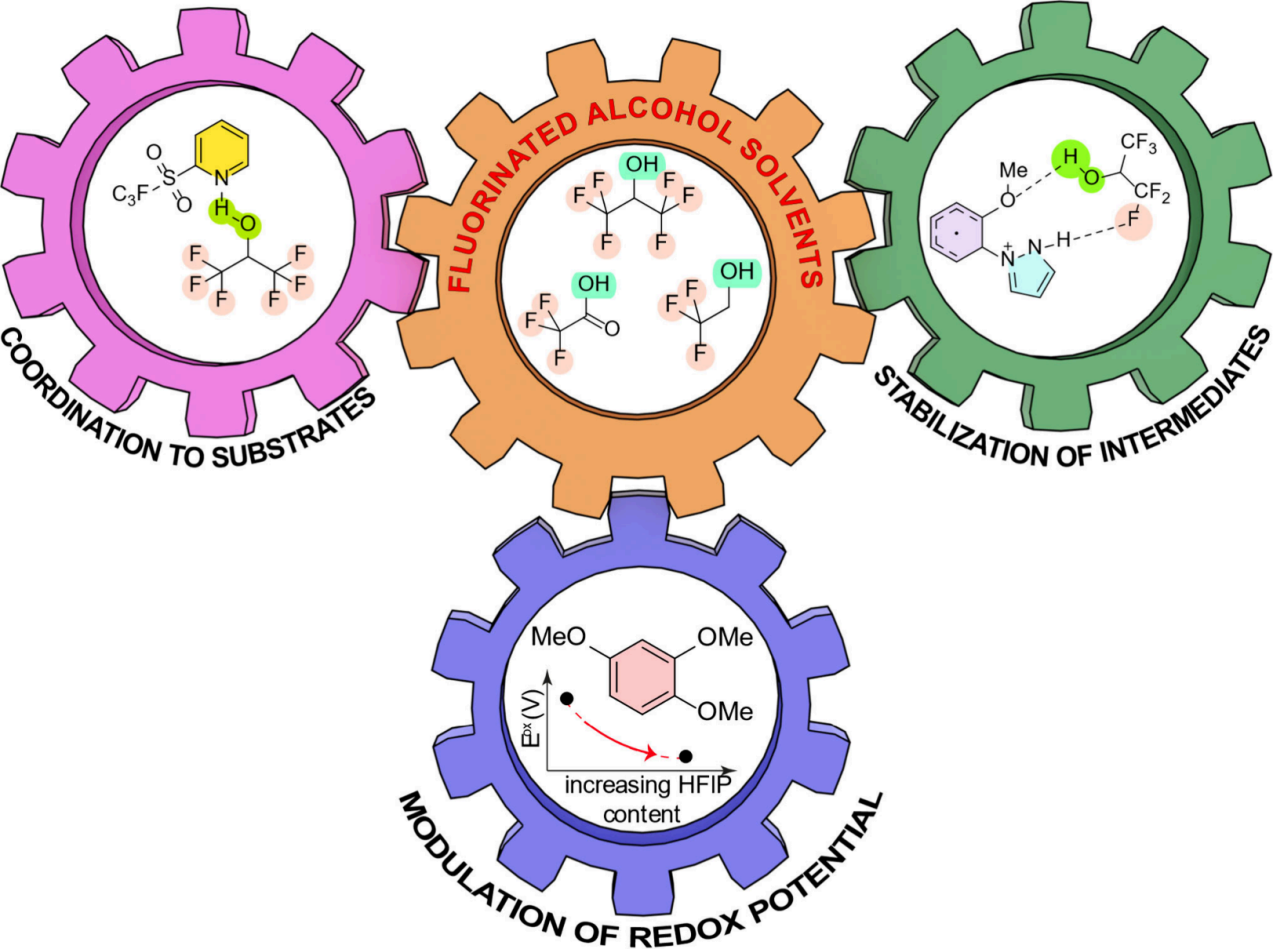
The many roles of fluorinated alcohols in organic electrosynthesis.

## Properties of Fluorinated Solvents

To understand the advantages of these fluorinated alcohol solvents as reaction media, the physical properties of the most representative examples will be discussed below. 1,1,1,3,3,3-hexafluoroisopropanol (HFIP) is more acidic in aqueous solution (pK_a_ = 9.3) compared to non-fluorinated alcohols, while it possesses a very reduced nucleophilicity, attacking only very reactive cationic species. Thus, HFIP is a very polar protic solvent but generally not a nucleophile, which can prevent side reactions from occurring. However, the main properties that explain the apparent supremacy of this solvent in electrosynthesis are its capability to stabilize radicals and its redox stability. The electrochemical domain of HFIP is comparable to that of acetonitrile (MeCN), with a potential window of about 4.6 V on glassy carbon (from −1.75 vs Ag/AgCl to 2.85 vs Ag/AgCl using tetrabutylammonium perchlorate as electrolyte), a value comparable to aprotic solvents, offering therefore the best of two worlds. On the other side, the acidity of HFIP may result in hydrogen evolution at negative polarizations.^18^ Nonetheless, HFIP has some drawbacks, like its toxicity and relatively high price when compared with other traditional solvents. However, its boiling point (59 °C) allows an easy recovery by simple distillation, reducing its economic cost and sustainability issues.^19,20^ Recently, mixtures of HFIP with tertiary amines have been described to act as electrolytes, which are able to provide enough ionic conductivity,^21^ in such a way this fluorinated solvent can be used as reaction media and electrolyte. Finally, it is worth noting that the high acidity of trifluoroacetic acid (TFA) pK_a(TFA)_ = 0.23 is not compatible with a broad range of functional groups in organic molecules, while its corrosive character is not desirable for large-scale applications. Therefore, using TFA as a solvent is usually not possible or implies important drawbacks. However, it has been employed as additive in electro-catalyzed reactions like the synthesis of benzazoles,^22^ benzimidazolones/benzoxazolones,^23^ the vicinal C–H amination,^24^ the hydroxyalkylation of *N*-heteroarenes,^25^ or the hydroarylation of alkynes with aryl iodides.^26^ In spite of all that, TFA has been used as neat solvent as well as solvent with MeCN, as will be discussed below.

Other fluorinated solvents have started to find applications in organic synthesis space but are still quite scarce, and no reports on their use in electrosynthesis have been found yet. Thus, this mini-review will primarily focus on the recent advances in electrosynthesis using HFIP, 2,2,2-trifluoroethanol (TFE) and TFA.

Similarly, 2,2,2-trifluoroethanol (TFE) possesses a lower boiling point (74 °C) compared to its non-fluorinated counterpart, and a higher density (1.38 g/mL). In addition, the strong negative inductive effect of the fluorine atoms makes the fluorinated alcohol more acidic [pK_a(ethanol)_ = 15.17, pK_a(2‑fluoroethanol)_ = 14.42, pK_a(TFE)_ = 12.4] (Table 1). Moreover, TFE has also a high ionizing power and relatively low nucleophilicity.^27^

**Table 1:** Fluorinated Solvent Properties Summary

Entry	Solvent	pK_a_	Boiling points (°C)	Vapor pressure (mmHg)
1	HFIP	9.3	59	159
2	TFE	14.42	78	71
3	TFA	0.23	72	110

## Fluorinated Alcohols As Non-innocent Reaction Media

The following examples illustrate how fluorinated alcohols can fulfill a dual role in organic electrocatalyzed transformations, acting as solvents but also as reagents.

The use of TFE as co-solvent in the 1,2-amino oxygenation of alkenes was recently reported by Lei *et al*.^28^ This approach is highly desirable for the straightforward synthesis of β-amino alcohols, which are important building blocks (Scheme 1). This reaction starts with the oxidation of the secondary amine derivative at the anode, affording an *N*-centered radical which is added to the alkene (Scheme 2). A second electron is then removed at the anode, affording a carbocation that is trapped by a nucleophile, while hydrogen is released at the cathode. Despite its relatively low nucleophilicity, TFE acts as a solvent but also as a nucleophile, being incorporated into the final product. However, the low nucleophilicity of TFE allows the incorporation of other nucleophiles. If carboxylates or pyrazole derivatives are added to the reaction media, these moieties are the ones reacting instead of the fluorinated alcohol. In contrast, performing the reaction in other solvents such as MeOH, EtOH or 2,2-difluoroethanol led to the incorporation of those solvents into the final product due to their higher nucleophilicity.

**Scheme 1:**
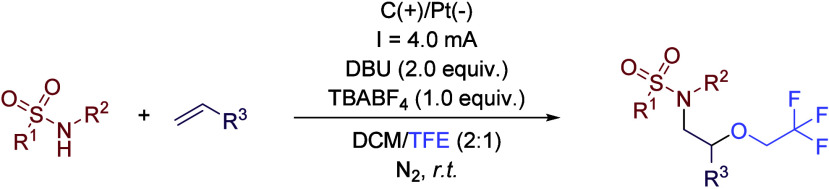
Electro-oxidative Alkene Oxygenation Adapted by Lei *et al.*

**Scheme 2:**
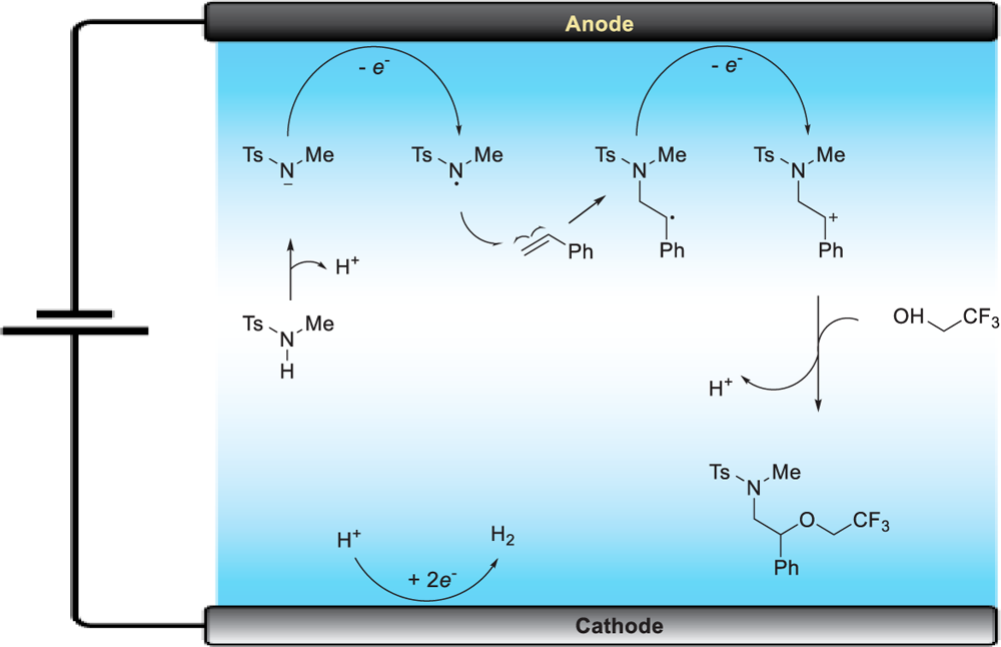
Proposed Mechanism of Alkene Oxygenation by Lei *et al.*

It is worth mentioning, that when either DCM or TFE were employed as neat solvents, only traces of the desired reaction product were obtained, while the mixture of both in 4:2 (DCM/TFE) ratio, afforded 76% yield. In this case, MeOH itself was a good solvent for the reaction, albeit in lower yield, while mixtures of DCM and MeOH afforded worse results than those obtained with DCM/TFE for the corresponding product (Table 2). Mixtures of fluorinated alcohols with solvents such as DCM are commonly explored, mainly to reduce the overall solvent cost,^29^ but in some cases like in this one, a synergistic effect between DCM and the fluorinated alcohol is observed, enhancing the reactivity, which can be attributed to the combined effect of intermediate stabilization and improved solubility of reagents. Fluorinated alcohols are known to enhance the lifetime of organic radicals, increasing the probability of the nitrogen-based radical to perform an addition to the styrene derivative, although the high polarity of this solvent may produce less polar compounds, like styrene, tending to form a separate liquid phase, which can be avoided by using a cosolvent like DCM.

**Table 2:**
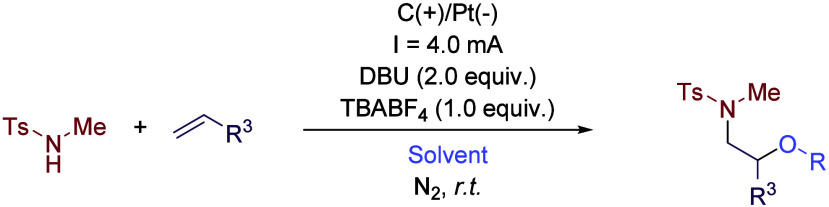
Solvent Comparison for the Reaction Depicted in Scheme 1

Entry	Solvent	Yield (%)
1	DCM/TFE (2:1)	76
2	DCM	traces
3	TFE	traces
4	HFIP	n.d.
5	DMC/MeOH (2:1)	40

Another case in which HFIP acts not only as a solvent, but also as a reagent was the functionalization of indoles and substituted anilines (Scheme 3).^30^ Using a reticulated vitreous carbon anode and a Pt plate as the cathode in the presence of TBAPF_6_ in a mixture of HFIP and DCM (4:3), the oxidation of the nitrogen-containing compound was hypothesized to give rise to a radical cation. This reacts with an *in situ* formed *O*-centered radical from HFIP, affording a cation intermediate, which subsequently reacts with a second molecule of HFIP. The reaction can also be performed in neat HFIP, but lower yields are then obtained, probably due to solubility issues.

**Scheme 3:**
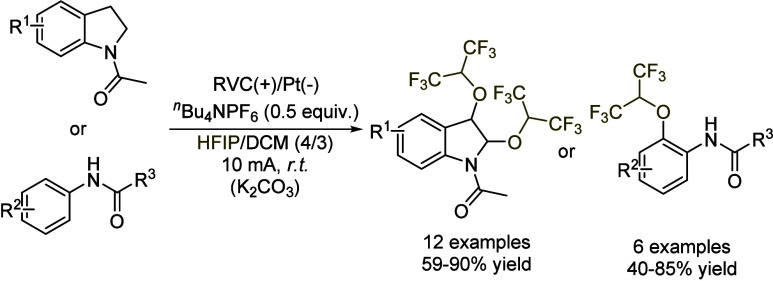
Indole/Aniline Functionalization by HFIP by Pan *et al.*

The next sections include reports involving the use of fluorinated alcohols without having a role as starting materials yet possessing important effects on the reaction outcome.

## C–H Functionalization Processes

C–H functionalization refers to a process in which a carbon–hydrogen (C–H) bond is cleaved, enabling the formation of new chemical bonds. Thus, by employing this strategy, C–H bonds are transformed to C–C or C-heteroatom bonds. It is noteworthy that due to the ubiquitous presence of C–H bonds in organic molecules, it represents one of the most useful transformations in organic synthesis, since pre-functionalization of substrates is avoided.^31^ This efficient approach not only reduces the number of synthetic steps required but also minimizes the generation of unwanted byproducts, contributing to greener and more sustainable synthetic routes and offering a new toolbox to obtain otherwise impossible to synthesize molecules.

## C–H Activation

When the C–H bond cleavage is mediated by the insertion of a transition metal catalyst, the process is defined as C–H activation. Regarding the general mechanism of these processes, after the C–H activation step, the product is released from the metal center, liberating the metal catalyst with a lower oxidation state than that at the initial step. Thus, a reoxidation of the catalyst is generally needed, which can vary greatly depending on the catalytic system, but common examples employ metallic salts like copper acetate or expensive silver derivatives.^32^ In this sense, the use of electrochemistry to perform this oxidation is highly desirable since no stoichiometric chemical oxidant is required, reducing the waste and the reaction cost.

Although HFIP has attracted the most interest as a solvent, TFE is also a versatile reaction medium that has found recent applications in organic electrosynthesis. In 2019, Ackermann and coworkers described a cobaltaelectro-catalyzed C–H/N-H activation protocol. During the optimization survey, authors did not observe any reactivity in solvents such as MeOH, MeCN, DMSO, DMF or H_2_O; however, the reaction occurred in HFIP and slightly better in TFE (Table 3).^33^ Although the authors did not comment any further on the solvent effect, clearly the use of these fluorinated solvents is crucial for the reaction outcome. After further optimization of reaction conditions, 30 examples were reported with yields up to 92% (Scheme 4). A possible explanation is that the use of these alcohols reduces the potential to perform the oxidation of the cobalt­(II) precatalyst, while improving the overall solubility and stability of reaction intermediates (Scheme 5).

**Scheme 4:**
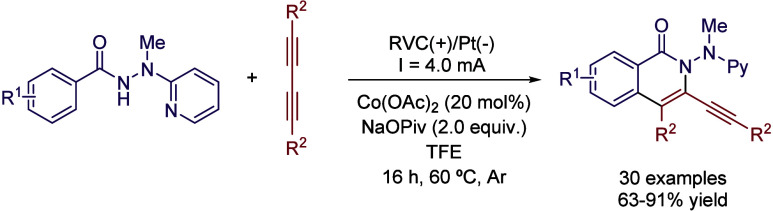
Cobaltaelectro-catalyzed C–H/N–H Activation by Ackermann *et al.*

**Table 3:**
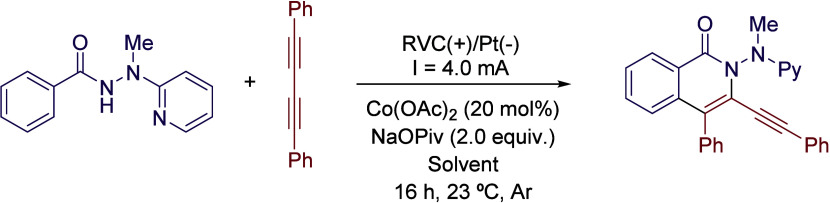
Solvent Comparison for the Optimization Process at 23 °C of the Reaction Depicted in Scheme 4

Entry	Solvent	Yield (%)
1	MeOH	trace
2	MeCN	n.d.
3	DMF	n.d.
4	DMSO	n.d.
5	H_2_O	n.d.
6	HFIP	30
7	TFE	34

**Scheme 5:**
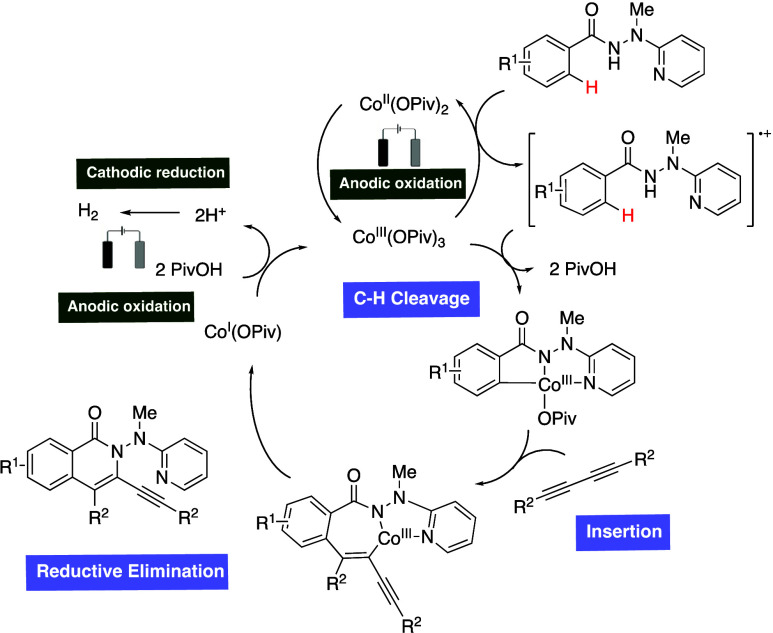
Reaction Mechanism of Cobaltaelectro-catalyzed C–H/N–H Activation by Ackermann *et al.*

More recently, the synthesis of pyrroles and lactones was described by Ackermann’s group via an electrocatalyzed C–H activation process (Scheme 6).^34^ A rhodium complex is employed in a solution of TFE with 3 equiv of NaOAc as a base without the need of further electrolytes. The reaction between disubstituted alkynes and several enamides is thus accomplished using a graphite felt (GF) as anode and a Pt plate cathode with a constant current of 1.5 mA. However, when the solvent system is switched to ^*t*^AmOH/H_2_O (3:1) a complete change of chemoselectivity is observed, and *N*-acyl substituted lactones are obtained instead of the aforementioned pyrrols.

**Scheme 6:**
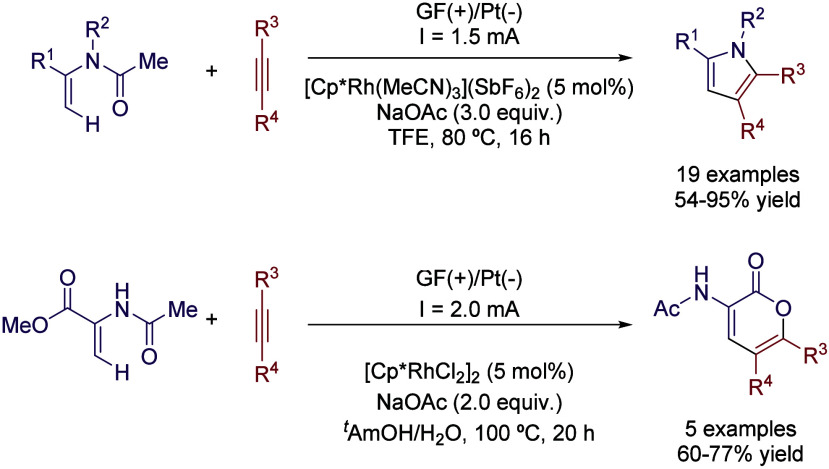
Rhodaelectro-catalyzed C–H Annulation by Ackermann *et al.*

Very recently, the same group reported the Pd-electrochemical C–H alkenylation of arenes.^35^ Unlike most Pd-catalyzed C–H activation strategies, the preinstallation of a directing group on the substrate was not necessary, reducing the number of synthetic steps and enabling the possibility of performing late-stage functionalization of complex molecules. The use of HFIP as co-solvent seemed to be mandatory once again, as the reaction performed in *N*-methyl-2-pyrrolidone/acetic acid (NMP/AcOH,1:2) or TFE:AcOH yielded almost no desired product. Different proportions of HFIP/AcOH were tested (from 1:1 to 1:4), with optimal results obtained in the mixture of 1:2 (Table 4). While the presence of AcOH could be explained by the need of an acidic media and the involvement of acetic acid/acetate in the palladium catalytic cycle, the role of HFIP as intermediate stabilizer becomes evident in view of the aforementioned experiments. Using this solvent system, a GF anode and a Pt plate cathode, Na­(OAc) as a base, and catalytic amounts of 1,4-benzoquinone, the catalytic system formed by Pd­(OAc)_2_ and 2-methyl-2-(phenylthio)­propanoic acid ligand, the coupling of a broad scope of arenes and acrylates was efficiently achieved (Scheme 7). Interestingly, unlike most of the set-ups described above, in this case a divided cell was employed, obtaining only traces if an undivided cell was used.

**Scheme 7:**
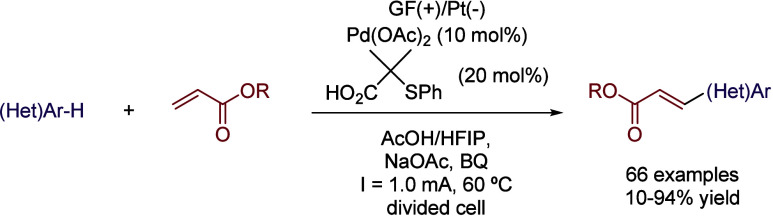
Undirected C–H Alkenylation of Arenes by Ackermann *et al.*

**Table 4:**
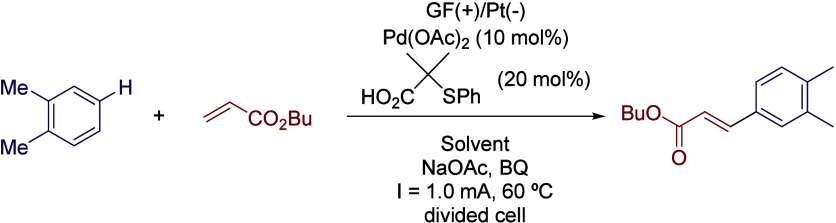
Solvent Comparison for the Optimization Process of the Reaction Depicted in Scheme 7

Entry	Solvent	Yield (%)
1	NMP:AcOH (1:2)	<5
2	TFE:AcOH	<5
3	HFIP:AcOH (1:1)	63
4	HFIP:AcOH (1:2)	76
5	HFIP:AcOH (1:3)	60
6	HFIP:AcOH (1:4)	60

## Heterogeneous C–H Functionalization

Other C–H functionalization processes have also been described using electrosynthetic methods without having a homogeneous metallic catalyst, such as the ones described in the C–H activation processes above. Due to their interest in pharmaceutical industry, the synthesis of *N*-aryl compounds has been deeply studied, with C­(sp^2^)–H/N–H cross-coupling reactions being very popular.^36^ After so much research in the field, these reactions are very efficient. However, traditional approaches make use of precious metal catalysts and stoichiometric amounts of chemical oxidants. In this sense, an electro-oxidative C–H amination of heteroarenes was reported, involving the use of Pt as anode and cathode and TBABF_4_ as electrolyte in a DCM:HFIP (1:1) solvent mixture.^37^ Note that DCM was required to solubilize the starting materials, whereas HFIP or TFE was critical to achieve significant yields of conversion. Likewise, it is worth noting that worse results were obtained with other mixtures, such as DCM/TFE, MeCN/HFIP, or MeOH/HFIP. Under optimized conditions, 2- or 3-subtituted indole derivatives were efficiently coupled with different anilines with moderate to excellent yields, with the procedure being extensible to other heterocycles like (benzo)­thiophene or (benzo)­furane derivatives (Scheme 8).

**Scheme 8:**
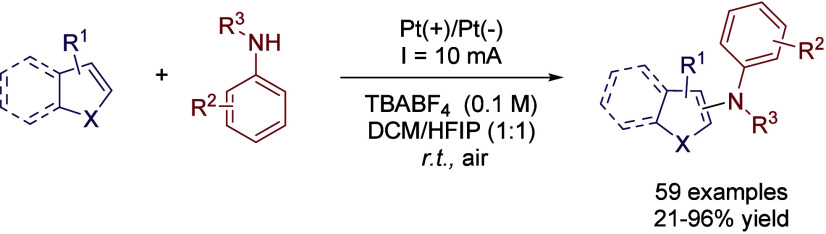
Heteroarene Electro-oxidative C–H Amination by Feng *et al.*

Moreover, other functional groups can be installed into aromatics via electrochemical transformations. The introduction of a trifluoromethoxy moiety in an aromatic ring can induce important effects on the bioavailability, metabolic stability, and lipophilicity, and other important parameters for a biologically active compound.^38^ Thus, the drug discovery sector is eager to find simple and efficient ways to perform this transformation. In this sense, a convenient and conceptually new process was described by Qing *et al.* in which an inexpensive trifluoromethylation reagent (trifluoromethyl 2-pyridyl sulfone) was employed and reduced at a platinum cathode to release a trifluoromethyl radical specie, which reacted with molecular oxygen to afford a F_3_COO radical, an unstable species that readily undergoes a second reduction to afford a F_3_CO radical. Finally, addition of this radical to the (hetero)­aromatic substrate and anodic oxidation and rearomatization afford the desired trifluoromethoxylated product. This reaction is performed in HFIP as the solvent, and the authors attribute many roles to the fluorinated alcohols to explain the success of the transformation. Firstly, HFIP coordinates to the nitrogen atom of the trifluoromethylation reagent via hydrogen bonding. This interaction is expected to facilitate SET (Single-Electron Transfer) reduction. In addition, HFIP increases the amount of oxygen in solution compared to other organic solvents and increases the lifetime of radical intermediates. With this simple procedure, up to 45 compounds were prepared with moderate to good yields, including examples of late stage functionalization of drug molecules (Scheme 9).^39^ Other examples reporting addition of electrogenerated radicals to heteroaryl molecules in fluorinated solvents are also found in the literature.^40–42^

**Scheme 9:**
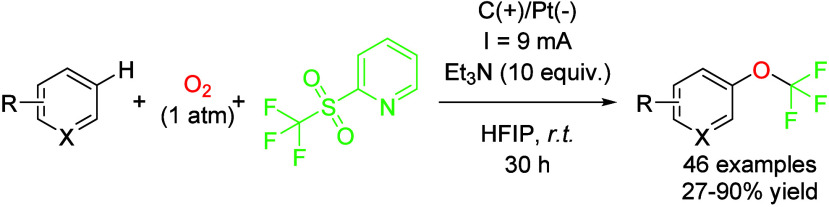
Trifluoromethoxylation of Heteroaromatic Compounds by Qing *et al.*

Benzylic positions are especially reactive and prone to be oxidized. In this sense, a mixture of DCE:HFIP (2:1) was proposed for the selective C­(sp^3^)-H bond fluorination.^43^ Instead of employing more established methods, like the use of electrophilic fluorinating reagents (*e.g.*, selectfluor), this approach provides a clean fluorination protocol under electrochemical conditions without the need of transition metal catalyst, chemical oxidants, or fancy reagents. By employing a cell with a graphite felt anode and a Pt plate cathode, benzylic C–H bonds are functionalized to C–F bonds by employing inexpensive NEt_3_·3HF as the fluorine source. During the optimization studies, MeCN was employed as solvent, leading to the desired product but yielding as well the acetamidation side-reaction due to the nucleophilic attack of the solvent. Therefore, HFIP was added as co-solvent with MeCN, using CsF as fluorine source, but in that case, the main product was the nucleophilic attack of the corresponding alkoxylate from the solvent. Results were slightly improved by changing the F^–^ source to NEt_3_·3HF (Scheme 10). After further optimization, the electrodes were changed, and the solvent system was modified to DCE:HFIP (2:1). Under these conditions, no competition of the solvent as nucleophile was observed, with the reaction cleanly yielding the desired product without by-products, while the same conditions with TFE instead of HFIP still offered the trifluoroethoxy-substituted by-product, due to the higher nucleophilicity of this solvent. Under the optimized conditions, moderate to excellent yields were obtained for different alkylbenzenes, with the reaction being compatible even with diarylmethylenes and the adamantane moiety.

**Scheme 10:**
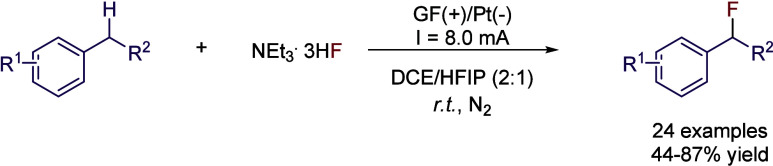
C­(sp^3^)-H fluorination by Ackermann *et al.*

It is worth noting that cyclic voltammetric studies revealed that addition of small amounts of HFIP to a substrate solution in DCE shifted the oxidation event to less positive potentials: from 2.16 V vs SCE in neat DCE to 1.73 V vs SCE in the mixture DCE:HFIP (2:1), with a second oxidation event appearing, which suggest that HFIP enables the generation of a stable radical intermediate.^44^ Instead of fluorination, similar strategies have been applied to other benzylic moieties to perform amination reactions under similar conditions.^45^

## Cross Dehydrogenative Coupling

Cross dehydrogenative coupling (CDC) is a synthetic methodology that consists of generating new C–C bonds by using only C–H bonds as reactants under oxidative conditions.^46^ This is achieved *via in situ* generated reactive intermediates. This concept was also expanded to the formation of carbon-heteroatom bonds,^47^ and as with other C–H functionalization processes described above, this approach bypasses the need for pre-functionalized starting materials, making it an efficient strategy for the construction of complex molecules.

The use of HFIP in organic electrosynthesis was pioneered by Waldvogel’s group. One of their early reports on this matter was the cross dehydrogenative coupling of phenols and naphthols using graphite electrodes,^48^ with improved results reported by the use of boron-dopped diamond electrodes.^49^ The synthesis of these biphenols via electrolysis is rather difficult since the homocoupling in a mixture of two different phenol derivatives is expected to be preferred over the cross-coupling reaction. However, solvent effect can modify the oxidation potential of individual substrates and achieve to break the direct relationship between nucleophilicity and oxidation potential. When having two aromatic systems, the one with higher electron density will be the most nucleophilic one, but at the same time the easiest to oxidize. Thus, under normal conditions, homocoupling is expected under electrolyzing conditions, but using HFIP as cosolvent allowed the selective formation of the cross-coupling product. Although the reason for this solvent effect is still unclear, authors claim that a change in nucleophilicity can be attributed to different solvation of the substrate by HFIP (Scheme 11).^50^ Therefore, selective cross-coupling reactions can be performed where only homocoupling would be obtained in other solvents.

**Scheme 11:**
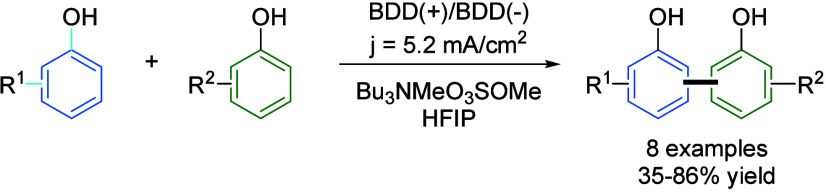
Cross Dehydrogenative Coupling of Phenols and Naphthols by Waldvogel *et al.*

This phenol homocoupling can be performed in a number of fluorinated solvents, including TFE and HFIP, but latter studies revealed that better yields and selectivities were obtained with TFA as solvent, taking advantage of the highly acidic reaction media.^48^

The same research group also employed phenols to functionalize benzothiophenes.^51^ Under conditions similar to those described above, 2- or 3- substituted benzothiophenes were efficiently coupled with different phenol derivatives with moderate to excellent yields (Scheme 12). The role of HFIP was again crucial to stabilize the intermediates formed at the anode while lowering the oxidation potential of phenols. This methodology was also successfully applied to the coupling of benzofuran derivatives with phenols, as well as with alkenes and alkynes.^52,53^

**Scheme 12:**
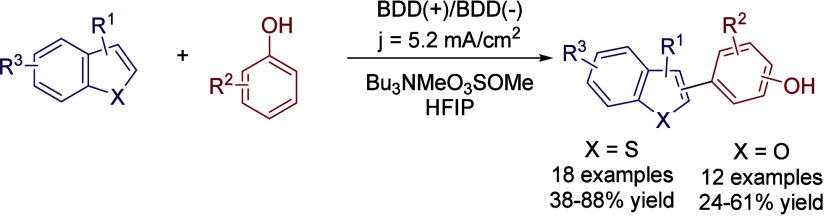
Electrocatalyzed Benzothiophene Functionalization by Waldvogel *et al.*

Regarding the synthesis of diaryl ethers, traditional methods involve cross-coupling reactions such as Ullmann or Buchwald-Hartwig transformations, which are catalyzed by transition metals. In this sense, an electrochemical approach was introduced to avoid the use of transition metals and stoichiometric oxidants in solution by performing an electro-oxidative cross dehydrogenative-coupling of phenols with tertiary anilines.^54^ After optimizing the reaction conditions, TBABF_4_ was employed as an electrolyte in a mixture of HFIP/DCM (6:4) with a carbon felt anode and a Ni plate cathode and a constant current of 5 mA in an undivided cell. Under these conditions, a 78% yield of the coupling product was obtained for the model reaction between aniline and 4-*tert*-butylphenol. However, the yield was halved by employing only HFIP and decreased to only 9% using neat DCM, showing the need of the mixed solvent. Other solvents such as MeCN did not promote the reaction at all (Table 5). In general, good yields were obtained for the coupling product with excellent regioselectivity toward the *para*-substitution (Scheme 13). Another recent electro-oxidative CDC strategy was applied to phenol and azole derivatives, resulting in C–O and C–N bond formation. Firstly, a diaryl ether scaffold is formed via an *O*-radical intermediate and a subsequently, electro-induced nucleophilic substitution between the *in situ* generated phenol derivatives and different azoles gave place to the corresponding polyarenes, which possessed fluorescence properties (Scheme 14).^55^

**Scheme 13:**
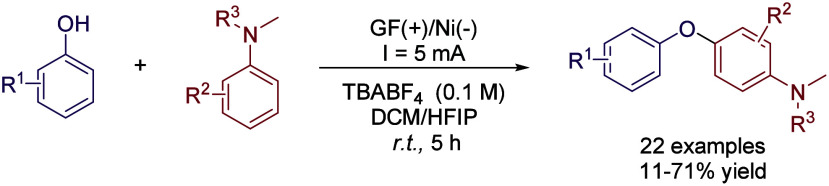
Electro-oxidative Cross Dehydrogenative-Coupling Of phenols by Vercammen *et al.*

**Table 5:**
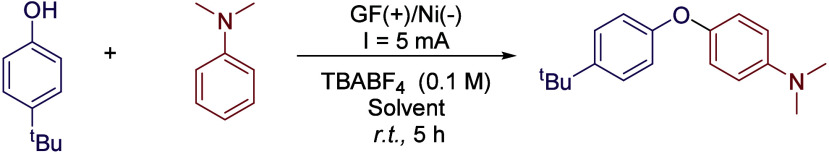
Solvent Comparison for the Optimization Process of the Reaction Depicted in Scheme 13

Entry	Solvent	Yield (%)
1	DCM/HFIP	78
2	DCM	9
3	HFIP	36
4	MeCN	n.d.

**Scheme 14:**
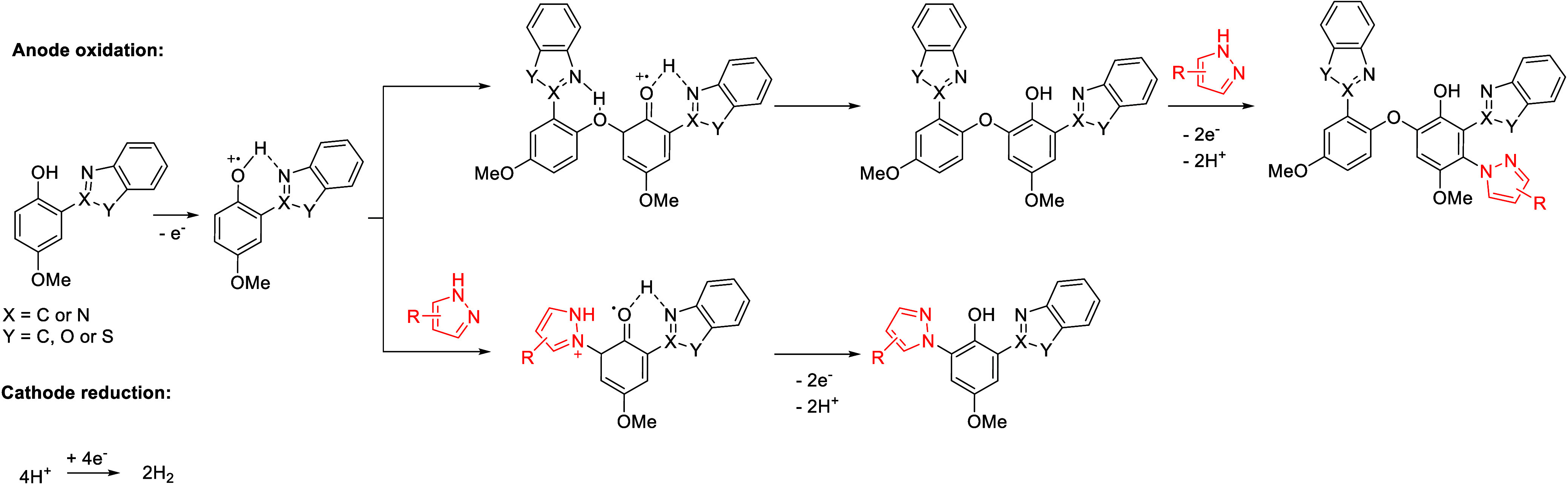
Proposed Reaction Pathway for the Electro-oxidative Cross Dehydrogenative Coupling by Feng *et al.*

The optimal reaction conditions were found to be using Pt as both the cathode and anode at a constant voltage of 2.5 V. In this case, TBABF_4_ was employed as the electrolyte and a mixture of DCM/HFIP (3:7) offered the best results. Lower reaction yields were obtained using DCM or DCE, while neat HFIP led to good conversion but lower than its mixture with DCM. Authors proposed that the reason for this enhanced reactivity in the solvent mixture might be due to a balance between the solubility of starting materials and the reactivity of the starting phenol derivative, which shows a lower oxidation potential in the mixed solvent compared to neat DCM (Scheme 15).

**Scheme 15:**
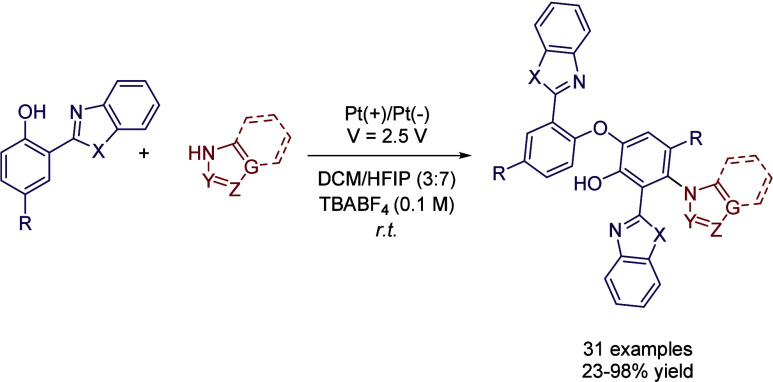
Electro-oxidative Cross Dehydrogenative Coupling by Feng *et al.*

Other cross coupling reactions involve C–S bond formation. In this sense, recent reports on the use of TFA as co-solvent include the continuous flow electrosynthesis of benzofused S-heterocycles *via* dehydrogenative C–S cross-coupling.^56^ This reaction is performed without the need of a supporting electrolyte by employing a continuous flow cell equipped with a Pt cathode and a carbon filled poly­(vinylidene fluoride) (C/PVDF) anode. A mixture of MeCN and TFA in a 9:1 ratio was employed as solvent with Sc­(OTf)_3_ acting as a catalyst. In this way, the intramolecular C–S cross dehydrogenative coupling of a variety of substrates was examined, affording moderate to good yields of 1,4-benzoxathiins and 1,4-benzothiazines bearing different substituents (Scheme 16). In addition to providing an acidic reaction media, one of the roles of TFA in the reaction is to increase the oxidation potential of the reaction product, avoiding the overoxidated by-product, as demonstrated by cyclic voltammograms (Figure 3).

**Scheme 16:**
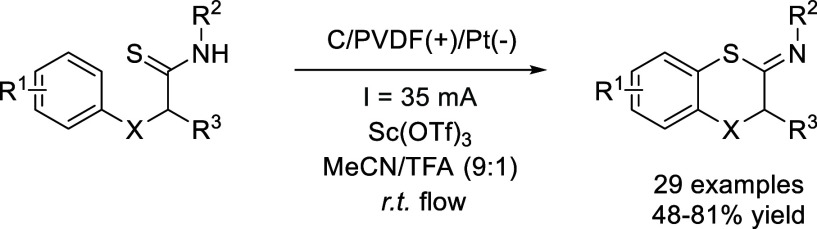
Dehydrogenative C–S Cross-Coupling by Xu *et al.*

**Figure 3:**
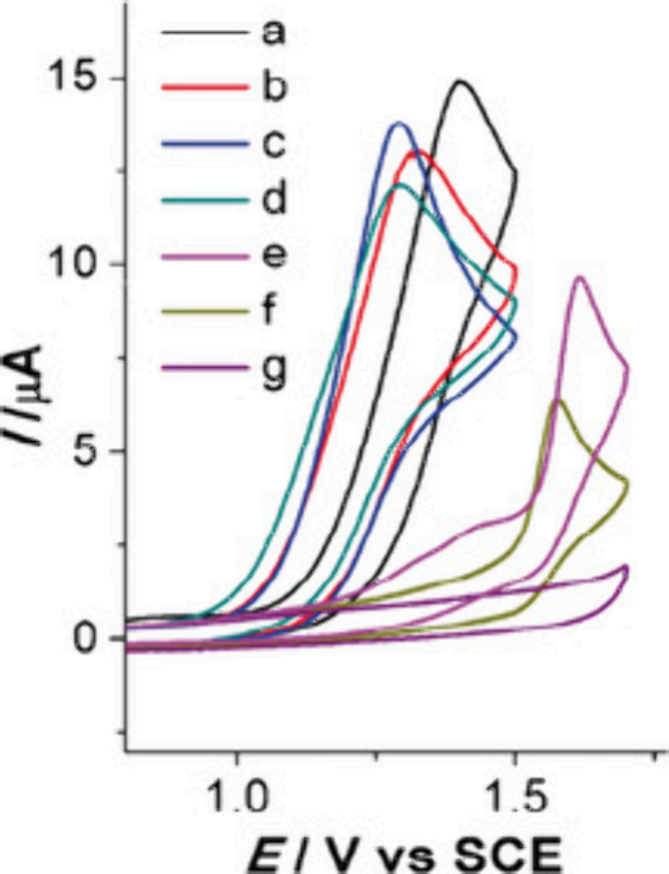
Cyclic voltammograms of *N*-isopropyl-2-phenoxypropanethioamide (**1**) and (*Z*)-*N*-isopropyl-2-methylbenzo­[*b*]­[1,4]­oxathiin-3­(2*H*)-imine (**2**). (0.1 M Et_4_NPF_6_). (a) **1** (3 mm), MeCN, E_p/2_ = 1.26 V; (b) **1** (3 mM), Sc­(OTf)_3_ (1 mM), MeCN, *E*_p/2_ = 1.18 V; (c) **1** (3 mM), MeCN/TFA (9:1), *E*_p/2_ = 1.18 V; (d) **1** (3 mM), Sc­(OTf)_3_ (1 mM), MeCN/TFA (9:1), *E*_p/2_ = 1.15 V; (e) **2** (3 mM), MeCN; (f) 2 (3 mM), Sc­(OTf)_3_ (1 mM), MeCN; g) **2** (3 mM), Sc­(OTf)_3_ (1 mM), MeCN/TFA (9:1). Reprinted (in part) with permission from ref 56. Copyright 2019 Wiley–VCH–Verlag GmbH & Co. KGaA.

CDC couplings can also be performed to generate new bonds between two different heteroatoms. In this sense, the synthesis of iminophosphoranes was described by the dehydrogenative P–N coupling of triarylphosphines with sulfonamides. This process was performed using a graphite anode and a stainless-steel cathode working in MeCN as solvent and tetraethylammonium iodide as the electrolyte. Authors thoroughly studied the effect of HFIP in the reaction, and after an exhaustive optimization, the use of the fluorinated additive was avoided in order to improve the sustainability standards of the process (Scheme 17). However, the best result was obtained by using a 0.15 M concentration of HFIP in the reaction media, which was explained by invoking the beneficial solvation properties and low nucleophilicity.^57^

**Scheme 17:**
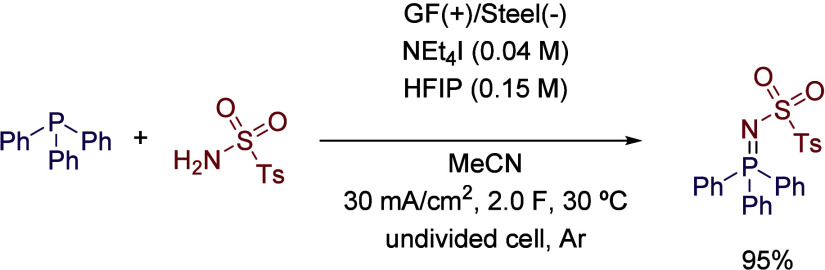
Dehydrogenative P–N Coupling Reaction by Waldvogel *et al.*

Other subtypes of coupling reactions have also been reported recently in fluorinated solvents. For example, the multicomponent synthesis of sulfonamides has been achieved via the generation of amidosulfinates from sulfur dioxide and secondary amines, followed by the addition to an electrochemically generated aryl radical.^58^ Sulfonamides have also shown to have interesting reactivity under electrochemical conditions in HFIP media, such as a migratory cyclization of *N*-acylsulfonamides, affording benzoxathiazine dioxides.^59^

## Synthesis of Heterocycles

Heterocycles are important motifs found in natural products and active pharmaceutical ingredients. This section summarizes some of the recent applications of electrosynthetic methods to prepare cyclic scaffolds containing oxygen and nitrogen atoms.

Firstly, it is worth highlighting the work from Pan and Lei’s group, who decided to study the electrochemical [3+2] annulation of styrene and acetylacetone without using transition metal salts or stoichiometric oxidants.^60^ The reaction was performed by using C and Pt electrodes in a mixture of MeCN and HFIP (3:1) with excellent yields. However, when the reaction was performed without HFIP or replacing it with other protic solvents such as water or methanol, no product was formed at all. Optimal conditions required 0.5 equiv of NaOAc as a base and 1.0 equiv of TBABF_4_ as supporting electrolyte. Under these conditions, several styrene derivatives and dicarbonyl compounds were successfully transformed to the corresponding dihydrofurans with moderate to excellent yields. The role of HFIP is thoroughly discussed by authors, who proposed the following mechanism: first, the dicarbonyl compound is deprotonated and oxidized at the anode, generating a radical which can be stabilized by the solvent. Then, addition to the styrene takes place, followed by a second oxidation, which yields the corresponding benzylic carbocation. Authors proposed then an addition of the HFIP alkoxide to this carbocation, which can promote an intermolecular nucleophilic attack to occur, releasing the product and HFIP (Scheme 18). This role of HFIP was demonstrated by replacing it by DMSO, which also has been reported to stabilize electrochemically generated carbocations,^61^ and the product was obtained, albeit in lower yields compared to when the fluorinated solvent was employed.

**Scheme 18:**
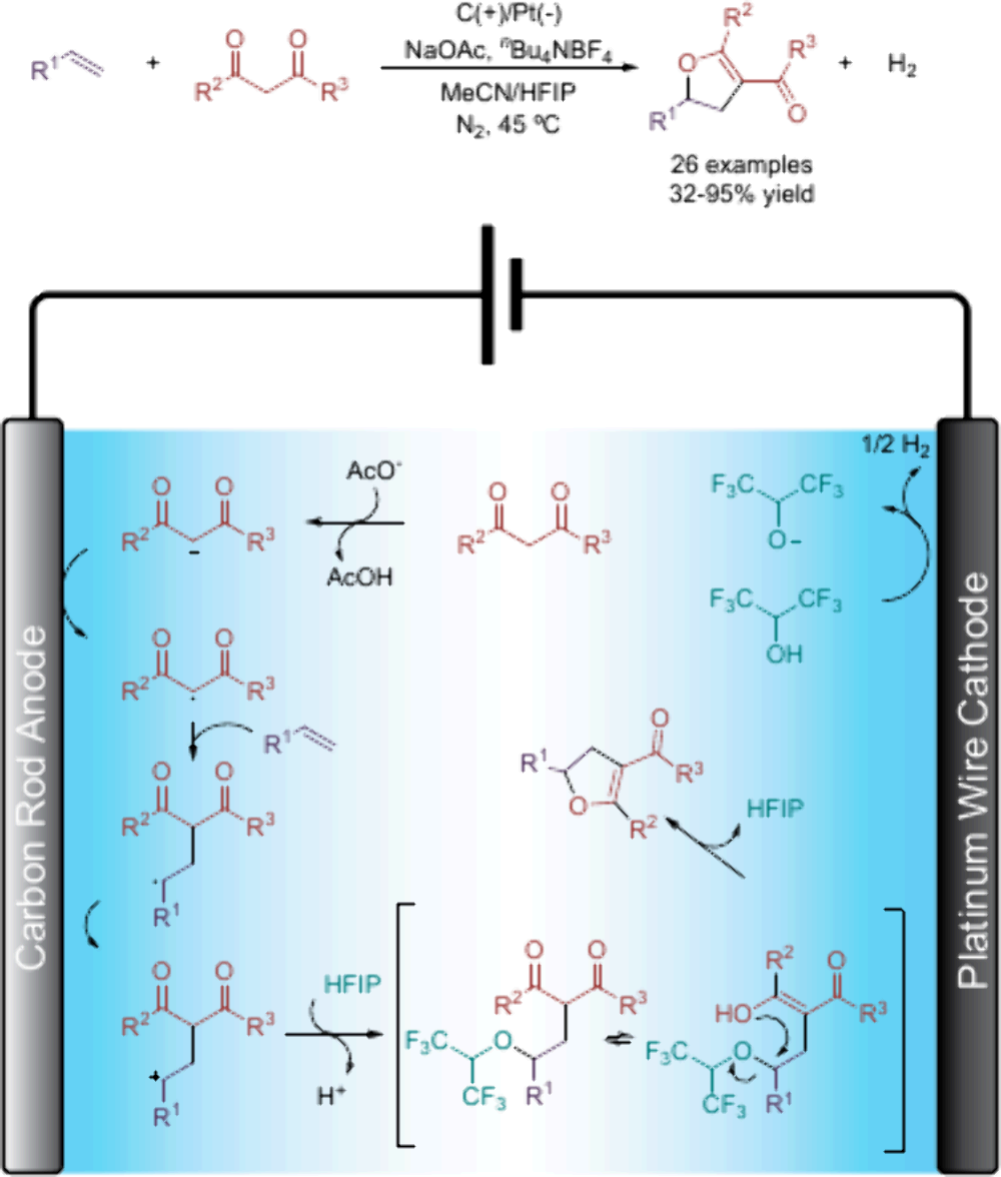
[3+2] Annulation of Styrenes and Dicarbonyl Compounds and Proposed Mechanism by Lei *et al.*

A similar approach was described for the synthesis of benzofurans by replacing styrenes by phenol derivatives.^62^ However, this approach involves the oxidation of the aromatic ring of the phenol to generate a phenoxonium cation, which can isomerize to form an oxonium Michael acceptor, which is nucleophilically attacked by the dicarbonyl compound. This intermediate can tautomerize to provide a phenol derivative, and finally an intermolecular condensation takes place assisted by HFIP and TBABF_4_, while the released protons are reduced at the cathode releasing H_2_. This reaction was performed in a DCM:HFIP mixture (1:1). Interestingly, removing HFIP resulted in a complete quenching of the reactivity, while performing the reaction without DCM decreased the reaction yield by only 5%. Optimal conditions involved the use of Pt plates as anode and cathode and TBABF_4_ as electrolyte at a constant potential between 2.3 and 2.7 V depending on the substrate. Good to excellent yields were obtained for *para*-substituted phenols bearing alkoxy- aryloxy- or even aryl substituents (Scheme 19). In addition, the applicability of the process was proven by synthesizing Dronedarone, a drug employed for the treatment of atrial fibrillation and atrial flutter.^63^

**Scheme 19:**
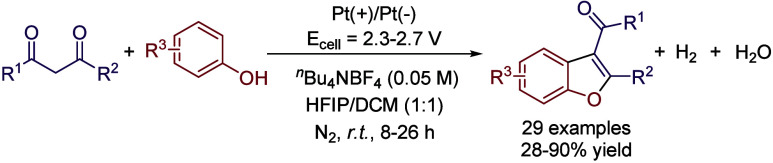
Electrosynthesis of Benzofurans by Kowey *et al.*

But indoles have not only been used as starting materials on the development of electrosynthetic methods, but also have been the target compounds based on 3+2 cycloaddition (Scheme 20).^64^ Even if the intramolecular cyclization of vinyl anilines has been more explored, reports on the more challenging intermolecular cyclization between anilines and 1,3-dicarbonyl compounds are scarce. The optimized method employs a graphite plate anode and a Pt anode under a constant current of 1.5 mA. The solvent of choice was neat HFIP, using TBABF_4_ as electrolyte and 30 mol % of trifluoromethanesulfonic acid as co-catalyst. DCM as the solvent nearly halved the reaction yield, and even worse results were obtained with MeCN (Table 6). The effect of the electrolyte was also capital, since the mere exchange of the TBA counterion from BF_4_^–^ to PF_6_^–^ reduced the reaction yield from 82 to 30%. The reaction was compatible with *N*-protected anilines bearing electrodonating groups and can be performed on gram scale with a slight decrease in the reaction yield.

**Scheme 20:**
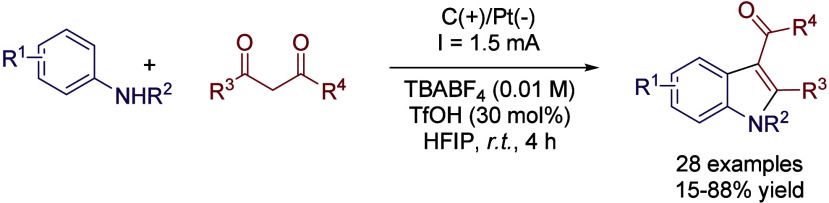
[3+2] Cycloaddition Indole Synthesis by Sun *et al.*

**Table 6:**
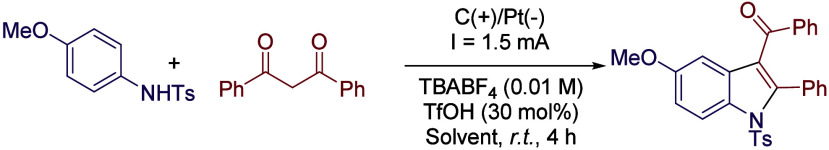
Solvent Comparison for the Optimization Process of the Reaction Depicted in Scheme 20

Entry	Solvent	Yield (%)
1	HFIP	82
2	DCM	45
3	MeCN	25

4-Imidazolidinones were also prepared by using a two-step strategy. First, the 4-component Ugi reaction was performed, affording an intermediate that was submitted to an electrocatalyzed cyclization.^65^ In this way, highly functionalized complex molecules were synthesized in just two steps from readily available simple molecules. The reaction, performed with a reticulated vitreous carbon (RVC) anode as a working electrode and a Pt plate cathode, yielded the spirocyclic 4- imidazolidinone by employing TBAPF_6_ as supporting electrolyte and HFIP as solvent (Scheme 21), while other solvents like MeOH or TFE or co-solvents such as dioxane, DCM or MeCN reduced the obtained yield (Table 7). HFIP is known to facilitate proton-transfer events through H-bonding and is the surveyed solvent which offered the best yield and has a lower pK_a_ value. Interestingly, HFIP was mostly recovered by distillation. Under optimized conditions, 36 examples of cyclization products were reported with moderate to good yields, constituting a greener approach for the synthesis of these complex molecules.

**Scheme 21:**
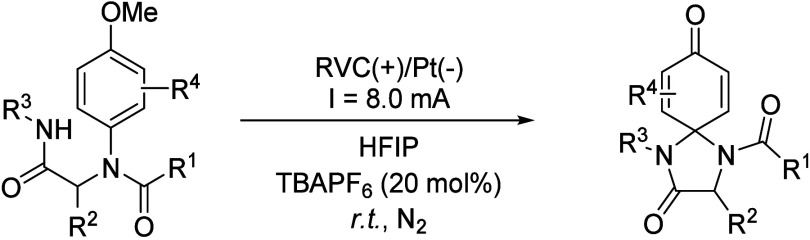
Electrocatalyzed 4-Imidazolidinones Synthesis by Sharma *et al.*

**Table 7:**
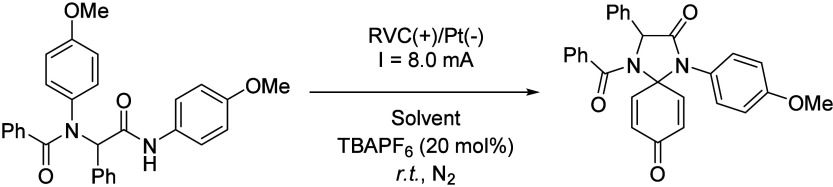
Solvent Comparison for the Optimization Process of the Reaction Depicted in Scheme 21

Entry	Solvent	Yield (%)
1	HFIP	74
2	DCM/HFIP (1:4)	50
3	Dioxane/HFIP (1:4)	42
4	MeCN/HFIP (1:4)	37
5	DMA/HFIP (1:4)	48
6	MeOH	0
7	TFE	24

Park and coworkers described the direct C_(sp3)_–H lactonization of 2-alkylbenzoic acids, synthetizing phthalides and other lactone derivatives (Scheme 22).^66^ The optimized reaction conditions involved the use of an undivided cell with a carbon anode and Ni cathode with a constant current of 15 mA in the mixture of DCM/HFIP and TBAClO_4_ as the supporting electrolyte. As with other examples on this review, solvent mixtures containing HFIP boosted the reaction yield by more than 3 times compared to neat DCM or other alcohol solvent. The authors invoked a reaction pathway wherein the HFIP plays an active role. As portrayed in Scheme 23, the reaction starts with (i) the oxidation of the carboxylate at the anode, followed by (ii) intramolecular hydrogen atom transfer (HAT) that generated a benzylic radical. This, (iii) is again oxidized to generate a carbocation that finally undergoes (iv) a cyclization releasing a proton. Meanwhile, at the cathode, protons present in the medium from (iv) or from HFIP are reduced to produce H_2_. Note that the latter renders the corresponding alkoxide, which could deprotonate the starting material closing the reaction pathway.

**Scheme 22:**
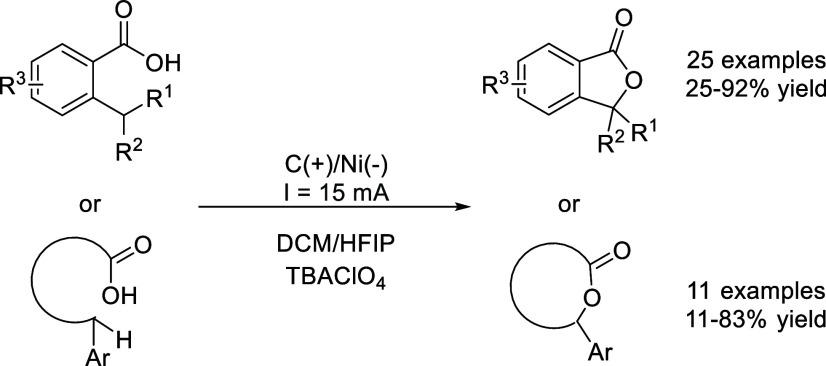
C_(sp3)_–H Lactonization by Park *et al.*

**Scheme 23:**
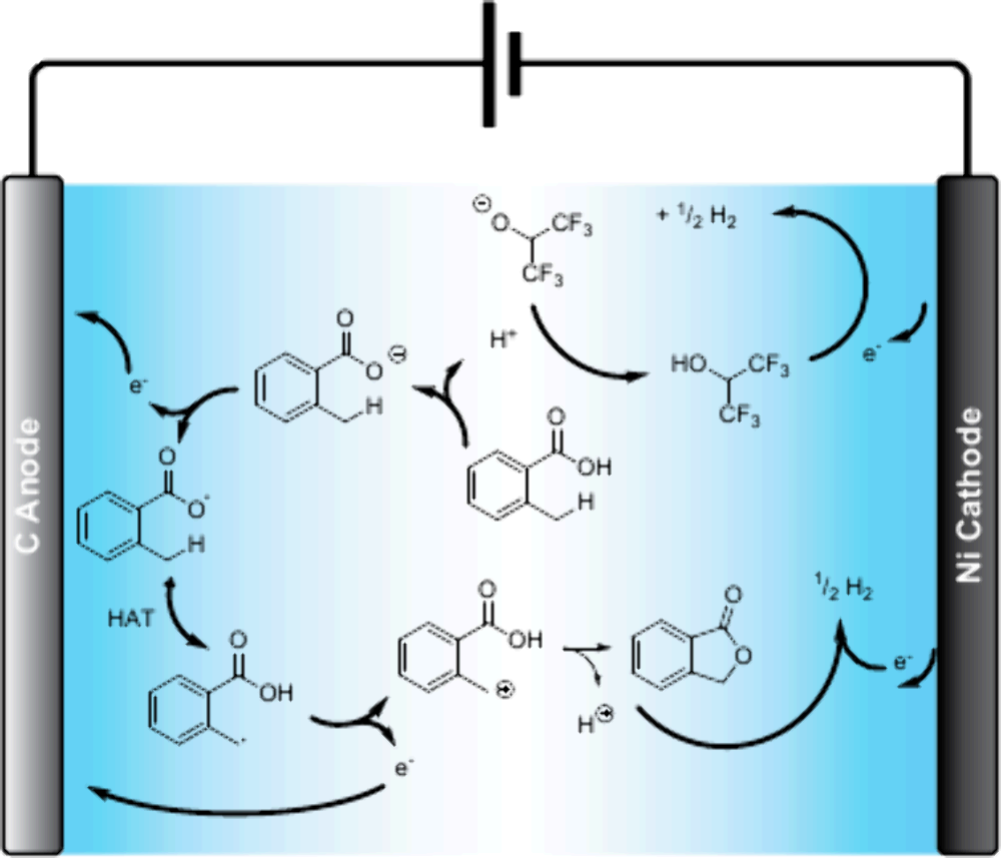
Proposed Mechanism for the Electrocatalyzed C_(sp3)_–H Lactonization by Park *et al.*

Finally, the synthesis of oxindole and quinolinone azide derivatives was achieved by employing a manganese-catalyzed electrochemical strategy.^67^ The reaction mechanism is based on the anodic oxidation of Mn^II^–N_3_ to a Mn^III^–N_3_ complex, which promotes the olefinic azidation that gives rise to a radical intermediate prone to suffer radical annulation. Afterwards, a second electron is yielded at the anode to generate a carbocation which suffers a deprotonative aromatization to afford the final product. This approach is performed using a graphite anode and a platinum foil cathode at room temperature in the presence of MnBr_2_ as catalyst, NaN_3_ as azidation reagent, and lithium perchlorate as supporting electrolyte in a mixture of MeCN and TFA. Again, the removal of TFA from the mixture led to a complete suppression of the reactivity, while other acids such as AcOH led to lower reaction yields. This improvement can be explained due to an enhanced solubility of all reagents as well as a better shuttle between electrodes. Under these conditions, 13 examples of substituted oxindoles and 10 examples of quinolinone were reported with moderate to good yields (Scheme 24). Azidocyanation of alkenes can also be performed under copper catalysis in HFIP as solvent.^68^

**Scheme 24:**
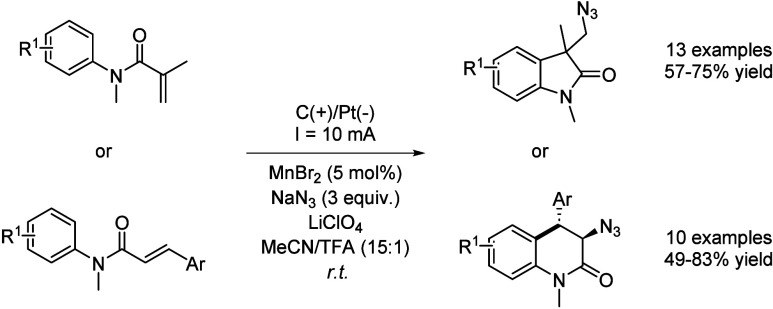
Manganese-Catalyzed Electrochemical Azidation-Annulation by Sarkar *et al.*

## Miscellaneous Reactions

This section covers some other reactions that do not belong to the previous sections. Recently, the continuous-flow synthesis of sulfur ylides via electro-oxidative coupling of sulfides with activated methylene compounds was reported. In this case, a mixture of DMSO and TFE (7:1) was employed as the reaction media. It is interesting to note that if TFE is replaced by TFA, no reaction is obtained, while other alcohols such as HFIP, EtOH, or MeOH afforded lower reaction yields (Scheme 25, Table 8).^69^

**Scheme 25:**
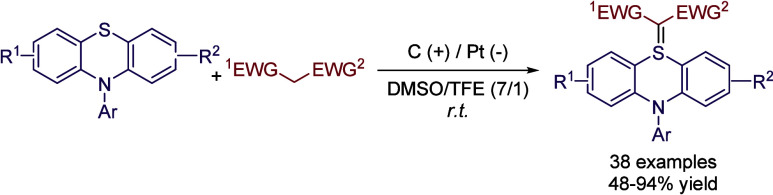
Electro-oxidative Synthesis of Sulfur Ylides by Guo *et al.*

**Table 8:**
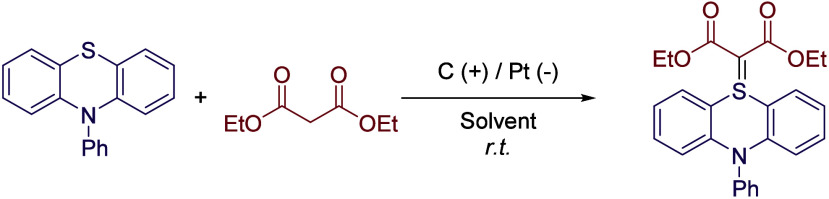
Solvent Comparison for the Optimization Process of the Reaction Depicted in Scheme 25

Entry	Solvent	Yield (%)
1	DMSO/TFE (7:1)	89
2	DMSO/TFA (7:1)	n.d.
3	DMSO/HFIP (7:1)	70
4	DMSO/EtOH (7:1)	60
5	DMSO/MeOH (7:1)	65
6	DMF/TFE (7:1)	40
7	MeCN/TFE (7:1)	37
8	DCE/TFE (7:1)	trace
9	Dioxane/TFE (7:1)	trace

Another interesting reactivity was reported by Hilt’s group, regarding the acyl nitroso Diels-Alder reaction of 1,3-dienes.^70^ The cycloaddition is achieved in high yields by using alternating current (AC), likely because operating in AC suppresses the commonly observed electrochemical decomposition of hydroxamic acids. During the optimization process, it was found that 20 mol % of HFIP in DCM was the optimal solvent mixture, since lower or higher volumes of HFIP led to a decrease in the reaction yield. The transformation was performed without the need of a supporting electrolyte employing 20 Hz AC, as the mixture of triethyl amine and HFIP served as the electrolyte. However, when working on direct current (DC) conditions, higher conductivity is required to obtain similar results, and therefore, an external supporting electrolyte and higher base concentration are mandatory. Interestingly, a sensitivity test was performed,^71^ showing that the reaction was not sensitive to inert conditions or applied current, while a large Pt electrode surface led to a great loss of reactivity (Scheme 26).

**Scheme 26:**
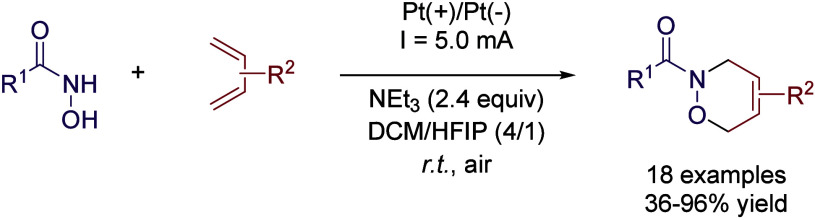
Acyl Nitroso Diels-Alder Reaction by Hilt *et al.*

Moreover, a surprising stereoselective electrosynthesis of α-diisoeugenol was reported with by combining HFIP as solvent and DBB electrodes.^72^ The reaction mechanism is initiated in a similar fashion to the ones previously described involving phenol moieties. However, the regioselective dimerization of isoeugenol can give rise to 4 different diastereoisomers, but only one was obtained following this protocol (Scheme 27). This unusual selectivity was attributed to HFIP, first due to the stabilization effect of radical species but also to the confinement of the radical on a solvation cage that induces a specific molecular orientation leading to the most favorable configuration.

**Scheme 27:**
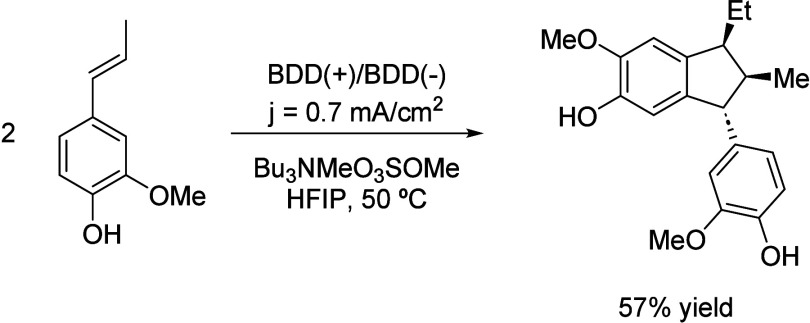
Selective Isoeugenol Dimerization by Einaga *et al.*

Organosulfur compounds are also important intermediates in organic synthesis. Thus, the activation of C–S bonds to access new C–C or C-heteroatom bonds has become an important field, but this strategy is typically based on the use of transition-metal catalysts or very activated substrates. However, the direct desulfurative electrooxidation of C­(sp^3^)-H bonds was recently reported. Thus, alkyl thiothers were subjected to a 5 mA current employing a carbon rod anode and a Pt cathode under nitrogen atmosphere, which *in situ* generated a benzylic carbocation that was trapped by a nucleophilic nitrogen-based heterocycle (including (benzo)­triazoles, (benzo)­pyrroles, or (benzo)­tetrazoles, among others, Scheme 28).^73^ These reactions were performed in MeCN as solvent with TBABF_4_ as electrolyte and using (4-BrPh)_3_N (10 mol %) as an organic redox mediator. The presence of HFIP was mandatory to boost the reaction yield, observing more than 20% yield drop without this co-solvent or by replacing it with *t*-BuOH. On the other hand, increasing the equivalents of HFIP did not result in a yield improvement. Although authors do not explain this effect any further, it is expected that 2.5 equiv of the fluorinated solvent helps to stabilize the radical intermediate, while higher amounts result in a total solvation of the cation and difficult nucleophilic attack.

**Scheme 28:**
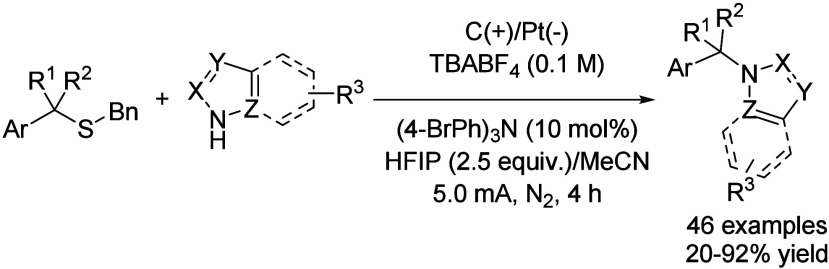
Desulfurative Electrooxidation C–N Coupling by Zheng *et al.*

Hypervalent iodine reagents have found plenty of applications in organic synthesis over the last few years.^74^ However, their isoelectronic Br (III) analogs have not been explored so much despite of their possibilities, and one of the main reasons is the difficulty of the synthesis.^75^ An important breakthrough in this field was the electrochemical approach for the synthesis of λ^3^-bromanes by anodic oxidation of aryl bromides bearing two *ortho*-hexafluoro-2-hydroxypropanyl substituents.^76^ This reaction was performed in HFIP at a constant current of 10 mA, while the reaction yield dropped when MeCN was employed as the solvent due to anodic degradation (Scheme 29). Interestingly, similar results were obtained in TFE and HFIP, but the latter was finally chosen due to its lower toxicity.

**Scheme 29:**
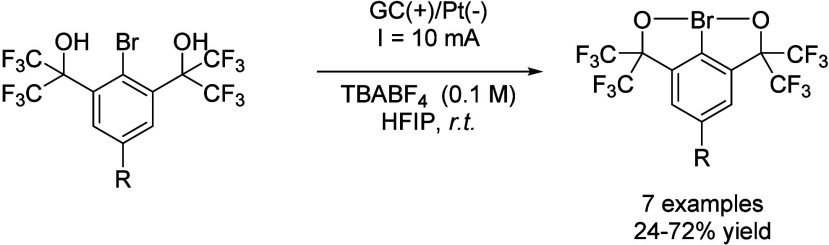
λ^3^-bromanes synthesis by Suna *et al.*

Another interesting case in this field is the synthesis of diaryliodonium salts, which are important electrophilic transfer reagents also employed as photosensitizers and as oxidants. The synthesis of these compounds typically involves the oxidation of iodoarenes to afford of λ^3^-iodanes followed by ligand exchange to afford stable salt compounds. This can also be done via electrolysis of 2-iodobiphenyls without external oxidants and in a one-pot, one-step manner.^77^ Thus, using a GC anode and a platinum cathode, 2-iodobiphenyl dissolved in a mixture HFIP/MeCN (4:1) with 5 equiv of triflic acid was subjected to a 5 mA constant current, affording the corresponding iodonium triflate. During the optimization process, TFE was employed as the solvent, causing a rapid increase in the cell voltage up to 30 V in a few minutes. Replacing TFE by HFIP increased the solution conductivity and therefore the desired product was obtained in 70% yield, with a cell voltage between 12 and 5 V. MeCN as solvent reduced the cell voltage while improving the yield up to 80%, but the best results, *i.e.*, 95% yield, were obtained in a mixture of HFIP/MeCN (4:1, Scheme 30, Table 9).

**Scheme 30:**
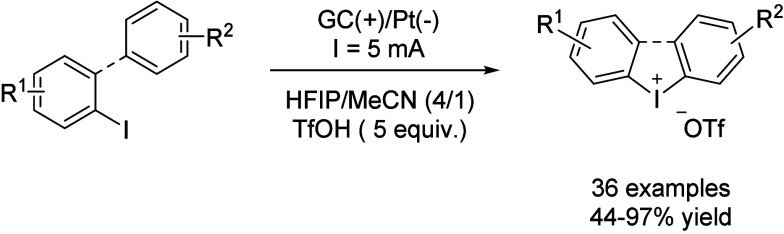
λ^3^-Iodane Synthesis by Moran *et al.*

**Table 9:**
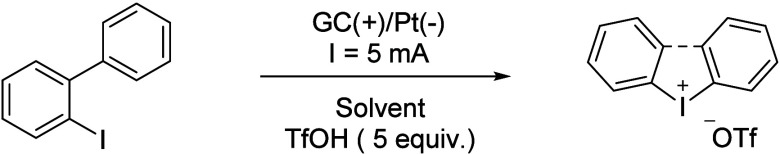
Solvent Comparison for the Optimization Process of the Reaction Depicted in Scheme 30

Entry	Solvent	Yield (%)
1	TFE	n.d.
2	HFIP	70
3	MeCN	80
4	DCM	33
5	EtOAc	30
6	TFE/MeCN (4:1)	83
7	HFIP/MeCN (4:1)	95
8	HFIP/MeCN (3:2)	87
9	HFIP/MeCN (1:1)	83

Good to excellent results were obtained with a variety of 2-iodobiphenyl derivatives, furnishing the corresponding cyclic iodane salts, but the procedure was also compatible with the use of iodobenzene derivatives in the presence of 1.5 equiv of arenes such as benzene, toluene, or mesitylene led to the formation of the acyclic diaryliodonium triflates.

The structural motif formed by the presence of vicinal dibromides or dichlorides has found many applications in compounds employed in the pharma industry as well as pest control agents or flame retardants. However, despite the great interest in some of these compounds, their synthesis is still quite hazardous. Another problem related to the use of these compounds is that some of them can be persistent pollutants non-biodegradable. In 2021, Dong *et al.* described an electron-shuttle process able to solve two problems at once (Scheme 31). On the one hand, dibromides or dichlorides found as environmental pollutants were employed as starting materials releasing an alkene, while other unsaturated compounds suffered the dehalogenation affording high-value compounds.^78^ This process was achieved by a paired electrolysis in which the cathodic reduction produced the bond cleavage of the halogenated pollutant, while the anodic oxidation allowed C–X bond formation. Although this process was performed in MeCN as the solvent, the addition of HFIP turned out to be crucial.

**Scheme 31:**
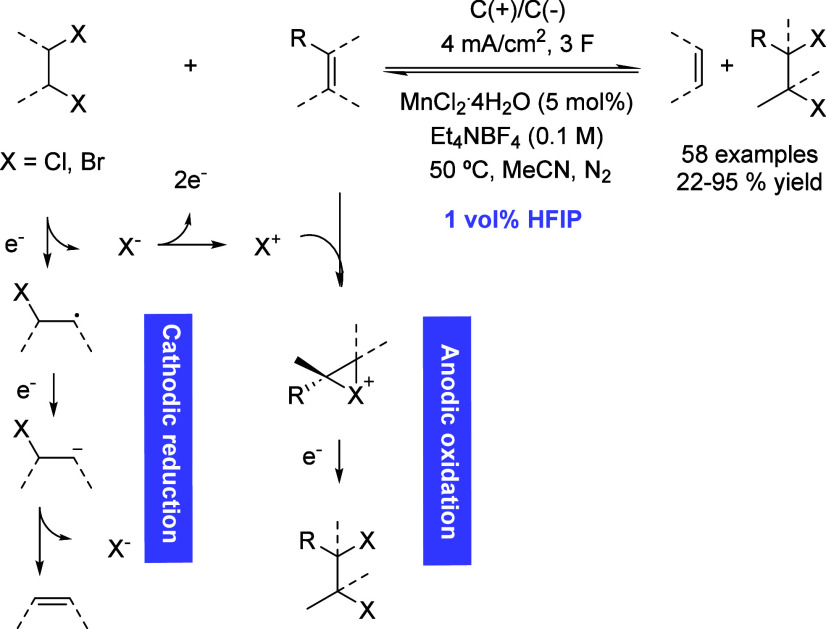
Electron Shuttle for Dehalogenation of Persistent Compounds by Morandi *et al.*

Cyclic voltammetry studies shed some light on this fact, proving that HFIP facilitated the reduction of dibromoethane by modifying its redox potential while at the same time suppressed the undesired reductive oligomerization.

Very recently, the combination of traditional enamine-based organocatalysis and electrochemistry has been reported, showing excellent yields and enantiomeric ratios by using a redox shuttle process,^79^ proving that new horizons regarding organocatalyzed electrochemistry are yet to be explored.

Most of the electro-catalyzed processes described herein are summarized in Table 10, including the reaction type, the solvent and electrodes employed, the supporting electrolyte (if any), and the range of yields obtained.

**Table 10:** Summary of the Reaction Conditions for the Representative Processes Discussed in This Work

Entry	Reaction	Solvent	Electrodes	Electrolyte	Yield range (%)
1	Alkene amino oxygenation	TFE/DCM	C(+)|Pt(−)	TBABF_4_	11–85 ^28^
2	Indole functionalization	HFIP/DCM	RVC(+)|Pt(−)	TBAPF_6_	40–85 ^30^
3	C–H activation	TFE	RVC(+)|Pt(−)	-^a^	63–91 ^33^
4	C–H activation	TFE	CF(+)|Pt(−)	-^b^	60–77 ^34^
5	C–H activation	HFIP/AcOH	GF(+)|Pt(−)	NaOAc	10–94 ^35^
6	C–H amination	HFIP/DCM	Pt(+)|Pt(−)	TBABF_4_	21–96 ^37^
7	C–H trifluoromethoxylation	HFIP	C(+)|Pt(−)	Et_3_N complex	27–90 ^39^
8	C–H fluorination	HFIP/DCE	GF(+)|Pt(−)	-^e^	44–87 ^43^
9	CDC phenols/naphthol	HFIP	BDD(+)|BDD(−)	Bu_3_NMeO_3_SOMe	35–86 ^49^
10	Benzothiophene-phenol coupling	HFIP	BDD(+)|BDD(−)	Bu_3_NMeO_3_SOMe	24–88 ^51^
11	CDC ArOAr’	HFIP/DCM	CF(+)|Ni(−)	TBABF_4_	11–71 ^54^
12	CDC phenols/azoles	HFIP/DCM	Pt(+)|Pt(−)	TBABF_4_	23–98 ^55^
13	C–S cross-coupling	TFA/MeCN	C/PVDF(+)|Pt(−)	-	48–81 ^56^
14	P–N dehydrogenative coupling	MeCN/HFIP	CG(+)|Stainles Steel (−)	NEt_4_I	95 ^57^
15	[3+2] cycloaddition-Dihydrofurans	HFIP/MeCN	C(+)|Pt(−)	TBABF_4_	32–95 ^60^
16	[3+2] cycloaddition-indoles	HFIP	C(+)|Pt(−)	TBABF_4_	15–88 ^64^
17	Benzofuran synthesis	HFIP/DCM	Pt(+)|Pt(−)	TBABF_4_	28–90 ^62^
18	4-Imidazolidones	HFIP	RVC(+)|Pt(−)	TBAPF_6_	52–85 ^65^
19	C_(sp3)_-H lactonization	HFIP/DCM	C(+)|Ni(−)	TBAClO_4_	11–92 ^66^
20	Azidation-annulation	TFA/MeCN	C(+)|Pt(−)	LiClO_4_	49–83 ^67^
21	Sulfur ylides	TFE/DMSO	C(+)|Pt(−)	TBAOAc	48–94 ^69^
22	Acyl-nitroso Diels-Alder	HFIP/DCM	Pt(+)|Pt(−)	-^c^	36–96 ^70^
23	Isoeugenol dimerization	HFIP	BDD(+)|BDD(−)	Bu_3_NMeO_3_SOMe	57 ^72^
24	λ^3^-bromanes	HFIP	GC(+)|Pt(−)	TBABF_4_	24–72 ^76^
25	λ^3^-iodanes	HFIP/MeCN	GC(+)|Pt(−)	-^d^	44–97 ^77^
26	Dehalogenation	HFIP/MeCN	C(+)|C(−)	Et_4_NBF_4_	22–95 ^78^

a 2 equiv of NaOPiv was employed as base.

b 3 equiv of NaOAc was employed as base.

c 2.4 equiv of Et_3_N was employed as base.

d 5 equiv of TfOH was employed as acid.

e 12 equiv of NEt_3_·3HF was employed as fluorinating reagent.

## Practicality of Fluorinated Alcohol Solvents

Although rarely discussed in the literature on electrosynthesis and organic synthesis, the use of fluorinated solvents comes along with severe environmental and biological threats. HFIP, TFE and TFA belong to the so-called perfluorinated and polyfluorinated alkyl substances (PFAS). Broadly speaking, PFAS encompass a very wide range of chemicals that continues to expand and that are key ingredients in the fabrication of fast-food containers, clothing, metal plating industry and semiconductor industry, amongst many others.^80^ One of the alarming issues with PFAS is related to its chemical stability, primarily derived from the inertness of the C–F bond. This accounts for the accumulation of PFAS in the soil, air, and water, promoting a persistent exposure of humans, wildlife, and the environment to these hazardous substances and, therefore, strengthening its deleterious effects. HFIP, arguably the most representative molecule among the fluorinated alcohols used in electrosynthesis, is corrosive and it is suspected to have potentially adverse effects on fertility, which certainly demands specific preventive measures when used.

The synthesis, cost, and recycling of fluorinated solvents are key parameters to be considered when assessing the viability of scaling these strategies. HFIP is prepared by catalytic hydrogenation of hexafluoroacetone.^81^ TFE is synthesized by catalytic hydrogenation of TFA, whereas TFA is produced by electrochemical fluorination of acetic anhydride.^81^ In all cases, fluorinated substrates prepared by reaction with hazardous HF are required as starting materials, which adds complexity to guarantee safety along the manufacturing process. The cost of, for instance, HFIP, is notably higher than common solvents due to not only the cost of the starting materials but also especially because of the synthetic process which involves dangerous chemicals, like HF, as well as purification treatments. As a result, putting in place recycling protocols to recover the solvent after the reactions is crucial to balance the high cost with the unique performance achieved with these solvents. In fact, leveraging the volatility of these compounds several authors have demonstrated that conventional distillation affords to recover HFIP after the reaction.^19,20,82^ However, given the almost infinite possibilities of formulating organic molecules, it would not be unreasonable to conceive the design of a new family of green solvents capable of mimicking the outstanding properties of fluorinated alcohol solvents.

With the rapidly growing notoriety gained by fluorinated alcohols as solvents, it becomes clear that more studies regarding long-term environmental contamination and life-cycle analyses must be conducted. However, the matchless results obtained with these solvents in some organic transformations will certainly encourage researchers to keep these solvents in mind when designing new processes.

## Summary and Outlook

Organic electrosynthesis has consolidated as a unique platform for chemical manufacturing, offering not only greener synthetic routes replacing reagents by electricity but also new reaction pathways originating from the distinct interactions occurring within electrochemical environments.

In this sense, tailoring the chemical properties of the solvents has been demonstrated to be a reliable strategy to manipulate the reactivity. Unlike conventional solvents that simply provide a liquid environment wherein the substrate, reagents, and electrolyte could dissolve and dissociate, the emerging family of fluorinated alcohol solvents has been shown to, additionally, actively interact with the substrates leading to new types of reactivity. These solvents have shown (i) to modulate the redox potential of the substrates and (ii) to strongly coordinate substrates and intermediate species, among others, thus impinging upon the regio- and chemoselectivity as well as upon the conversion yields. Indeed, these beneficial effects have been leveraged in a wide range of transformations, encompassing various C–H activation and cross-coupling reactions or the intra- and intermolecular synthesis of heterocycles, amongst others. All in all, this diversity of reactions supports the universality of this strategy and how custom-engineering solvents could afford tighter control over the synthetic route.

To date, there are only a couple of fluorinated alcohols, mainly HFIP and TFE, that have been exploited in electrosynthesis, mostly following the encouraging results gathered from conventional organic synthesis. But there is room for much exploration and discoveries.

On the one hand, new formulations of fluorinated alcohol solvents, such as 1,1,1-trifluoro-2-propanol (TFIP), hexafluoro-tert-butanol (HFTB) or 1-phenyl-2,2,2-trifluoroethanol (PhTFE), have displayed intriguing effects in organic synthesis but they have not been explored as solvents for electrosynthesis yet.^83^

On the other hand, understanding the requirements for the reaction could guide the design of optimum solvents wherein the polarizability, the H-bonding strength, or the coordinating characteristics could be programmed to maximize the interplay between the solvent and the reaction media. Following on the idea of improving the solvent characteristics, there are a few examples of organic electrosynthesis performed using ionic liquids or deep eutectic solvents to leverage their high conductivity,^84,85^ high boiling point and recycling possibilities amongst others. Finding new compositions capable of integrating fluorinated alcohol molecules could certainly bring the best of two worlds to a unique family of electrolytes.
